# CD157 Confers Host Resistance to Mycobacterium tuberculosis via TLR2-CD157-PKCzeta-Induced Reactive Oxygen Species Production

**DOI:** 10.1128/mBio.01949-19

**Published:** 2019-08-27

**Authors:** Qianting Yang, Mingfeng Liao, Wenfei Wang, Mingxia Zhang, Qi Chen, Jiubiao Guo, Bin Peng, Jian Huang, Haiying Liu, Ayano Yahagi, Xingzhi Xu, Katsuhiko Ishihara, Andrea Cooper, Xinchun Chen, Yi Cai

**Affiliations:** a Guangdong Key Lab for Diagnosis &Treatment of Emerging Infectious Diseases, Shenzhen Third People’s Hospital, Shenzhen University School of Medicine, Shenzhen, China; b Department of Pathogen Biology, Guangdong Key Laboratory of Regional Immunity and Diseases, Shenzhen University School of Medicine, Shenzhen, China; c Department of Cellular Biology, Shenzhen University School of Medicine, Shenzhen, China; d Key Laboratory of Systems Biomedicine (Ministry of Education), Shanghai Center for Systems Biomedicine, Shanghai Jiao Tong University, Shanghai, China; e Collaborative Innovation Center of Systems Biomedicine, Shanghai Center for Systems Biomedicine, Shanghai Jiao Tong University, Shanghai, China; f MOH Key Laboratory of Systems Biology of Pathogens, Institute of Pathogen Biology, Chinese Academy of Medical Sciences and Peking Union Medical College, Beijing, China; g Center for Tuberculosis Research, Chinese Academy of Medical Sciences and Peking Union Medical College, Beijing, China; h Department of Immunology and Molecular Genetics, Kawasaki Medical School, Kurashiki, Okayama, Japan; i Leicester Tuberculosis Research Group, Department of Infection, Immunity and Inflammation, University of Leicester, Leicester, United Kingdom; Max Planck Institute for Infection Biology

**Keywords:** CD157, *Mycobacterium tuberculosis*, PKCzeta, TLR2, reactive oxygen species

## Abstract

Recruitment of monocytes to the infection site is critical for host resistance against Mycobacterium tuberculosis. CD157 has a crucial role in neutrophil and monocyte transendothelial migration and adhesion, but its role in tuberculosis (TB) is unclear. Here, we show that both mRNA and protein levels of Cd157 are significantly increased during M. tuberculosis infection. Deficiency of Cd157 impaired host response to M. tuberculosis infection by increasing bacterial burden and inflammation in the lung in the murine TB model. *In vitro* experiments show that the bactericidal ability was compromised in *Cd157* knockout (KO) macrophages, which was due to impaired M. tuberculosis-induced reactive oxygen species (ROS) production. We further reveal that CD157 interacts with TLR2 and PKCzeta and facilitates M. tuberculosis-induced ROS production in *Cd157* KO macrophages, which resulted in enhanced M. tuberculosis killing. For the clinic aspect, we observe that the expression of CD157 decreases after effective anti-TB chemotherapy. CD157 is specifically increased in pleural fluid in tuberculous pleurisy patients compared to pneumonia and lung cancer patients. Interestingly, the levels of soluble CD157 (sCD157) correlate with human peripheral monocyte-derived macrophage bactericidal activity. Exogenous application of sCD157 could compensate for macrophage bactericidal ability and restore ROS production. In conclusion, we have identified a novel protective immune function of CD157 during M. tuberculosis infection via TLR2-dependent ROS production. Application of sCD157 might be an effective strategy for host-directed therapy against TB in those with insufficient CD157 production.

## Abstract (precis)

Tuberculosis, a chronic bacterial disease caused by Mycobacterium tuberculosis, remains a major global health problem. CD157, a dual-function receptor and β-NAD^+^-metabolizing ectoenzyme, promotes cell polarization, regulates chemotaxis induced through the high-affinity fMLP receptor, and controls transendothelial migration. The role of CD157 in TB pathogenesis remains unknown. In this study, we find that both mRNA and protein levels of CD157 are significantly increased in TB. Deficiency of CD157 impaired host defense against M. tuberculosis infection both *in vivo* and *in vitro*, which is mediated by an interaction among CD157, TLR2, and PKCzeta. This interaction facilitates M. tuberculosis-induced macrophagic ROS production, which enhances macrophage bactericidal activity. Interestingly, the sCD157 level in plasma is reversibly associated with MDM M. tuberculosis killing activity. By uncovering the role of CD157 in pathogenesis of TB for the first time, our work demonstrated that application of soluble CD157 might be an effective strategy for host-directed therapy against TB.

## INTRODUCTION

Tuberculosis (TB) is a major global public health issue and is responsible for the loss of 1.7 million lives annually (1). Macrophages are the most abundant host cell type found at sites of Mycobacterium tuberculosis infection and are implicated in disease control and progression depending, in part, on the macrophage lineage (2, 3). M. tuberculosis infection triggers monocyte recruitment from bone marrow and peripheral blood. Early study showed that lung interstitial macrophages derived from monocytes recruited during M. tuberculosis infection constrain bacterial growth, whereas alveolar macrophages provide a permissive environment for M. tuberculosis replication (3). Therefore, monocyte recruitment to the site of infection, such as the lung, is critical for host defense against M. tuberculosis. Clinical observations have also associated TB with defective monocyte migration (4). Consistently, data derived from a congenic monocyte adoptive transfer model showed that monocytes recruited from peripheral blood can differentiate into macrophages and dendritic cells that function in innate or adaptive immunity to fight M. tuberculosis infection (5). However, accumulating data suggest that excessive inflammation driven by uncontrolled neutrophil recruitment is also detrimental to the host during M. tuberculosis infection (6, 7). Therefore, immune cell migration to the lungs and lymph nodes should be appropriately regulated to ensure immunological benefit during M. tuberculosis infection.

CD157/BST1 is a dual-function receptor and β-NAD^+^-metabolizing ectoenzyme of the ADP-ribosyl cyclase family (8–10). Human CD157 is constitutively expressed by peripheral blood mononuclear cells (PBMCs) as well as synovial, vascular endothelial, mesothelial cells, and follicular dendritic cells. Through high-affinity binding to selected components of the extracellular matrix, CD157 has a crucial role in neutrophil and monocyte transendothelial migration and adhesion (9–12). By binding to scrapie-responsive gene 1 (SCRG1), CD157 plays an important role in regulating the stem nature and migration of mesenchymal stem cells (13). In addition, CD157 expressed by epithelial ovarian cancer cells and pleural mesothelioma cells controls tumor cell migration and invasion (14, 15). In addition to existing as a glycosylphosphatidylinositol (GPI)-anchored membrane protein implicated in the control of leukocyte trafficking, CD157 also presents as a soluble protein whose biological role is unknown (16, 17).

Considering the critical roles of CD157 in controlling cell migration, we hypothesized that CD157 might contribute to the host defense against M. tuberculosis infection by regulating leukocyte migration to the site of M. tuberculosis infection. To test this hypothesis, we profiled CD157 expression during M. tuberculosis infection in human TB and a murine TB model and further determine the role of CD157 in TB pathogenesis and its underlying mechanisms.

## RESULTS

### Cd157 expression is significantly increased in TB.

**FIG 1:**
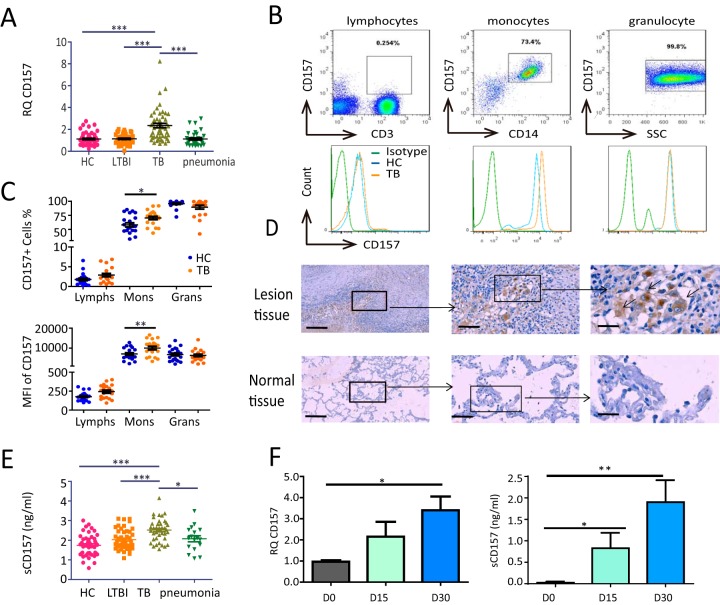
CD157 expression levels are significantly increased in patients with TB. (A) *Cd157* expression levels in whole blood from HC subjects (*n* = 55), LTBI subjects (*n* = 46), TB patients (*n* = 51), and pneumonia patients (*n* = 38) were determined by quantitative PCR (qPCR). RQ, relative quantification. (B) Peripheral blood was stained with anti-CD3, anti-CD14, and anti-CD157 antibodies and analyzed by flow cytometry. The histograms show the percentage of CD157-positive cells in lymphocytes, monocytes, and granulocytes from peripheral blood. SSC, side scatter. (C) The percentage (top panel) and mean fluorescence intensity (MFI) (bottom panel) of CD157 expression on lymphocytes (Lymphs), monocytes (Mons), and granulocytes (Grans) from HC subjects (*n* = 21) and TB patients (*n* = 20). (D) Immunohistochemical analysis of CD157 expression in tuberculous granuloma of lung tissue samples from patients with active tuberculosis (top panels) and in normal lung tissue adjacent to the lesions from patients with TB (bottom panels). Brown-labeled cells (arrow) represent CD157-positive cells. The images of the whole microscope slides were captured using a NanoZoomer digital pathology system (Hamamatsu Photonics). Outlined areas in the main images are enlarged in the insets. The bars represent 100 μm. Images that were representative of the images from three experiments were presented. (E) The concentrations of sCD157 in plasma from HC subjects (*n* = 46), LTBI subjects (*n* = 46), TB patients (*n* = 40), and pneumonia patients (*n* = 17) were determined by ELISA. (F) *Cd157* expression levels over time (days 0, 15, and 30) in the lungs of WT mice infected with strain H37Rv were determined by quantitative PCR (left). The protein levels of sCD157 in plasma from WT mice during M. tuberculosis infection determined by ELISA (right). One-way ANOVA Newman-Keuls multiple comparison test (A, E, and F) or unpaired *t* test (C) were used. Values are means ± standard errors of the means (SEM) (error bars). Values that are significantly different are indicated by bars and asterisks as follows: *, *P* < 0.05; **, *P* < 0.005; ***, *P* < 0.001.

Although infection with M. tuberculosis is the first step in the development of TB, the nature of the host resistance to M. tuberculosis infection plays a critical role in determining the outcome of infection and development of disease (18). Accordingly, if CD157 were important for host defense against TB, a difference in CD157 expression levels between subjects with latent tuberculosis infection (LTBI) and TB might be expected. To test this, we first compared the expression of CD157 among healthy controls (HC), subjects with LTBI, patients with TB, and patients with pneumonia. We found that CD157 mRNA expression was significantly increased in whole blood obtained from patients with TB compared to HC subjects, LTBI subjects, and patients with pneumonia (Fig. 1A).

Flow cytometric analyses indicated that CD157 is highly expressed on peripheral monocytes and neutrophils but that it is selectively increased on CD14^+^ monocytes in patients with TB compared to HC (Fig. 1B and C). In addition, we observed high expression of CD157 in human lung tissues from patients with TB (Fig. 1D). In addition to the membrane-bound CD157, we noted that the levels of soluble CD157 (sCD157) were significantly increased in plasma from TB patients compared to HC, LTBI, and pneumonia patients (Fig. 1E). Together, these data indicate that membrane-bound CD157 expression on monocytes and sCD157 levels in plasma are significantly increased in active TB, suggesting an association between CD157 expression and TB development.

### *Cd157* knockout mice are susceptible to M. tuberculosis infection.

CD157 plays an important role in regulating monocyte transmigration (9, 11, 12). Together with our finding that CD157 is selectively increased on monocytes of patients with TB, we investigated the role of CD157 in TB pathogenesis. We first determined whether increased CD157 expression is caused by a virulent M. tuberculosis strain H37Rv in an animal model. We found that CD157 mRNA expression levels in the mouse lung gradually increase over the course of M. tuberculosis infection (Fig. 1F). Consistent with our clinical observations, the levels of sCD157 also increase in the plasma of mice during M. tuberculosis infection (Fig. 1F). We thus concluded that increased CD157 expression is likely driven by M. tuberculosis infection.

**FIG 2:**
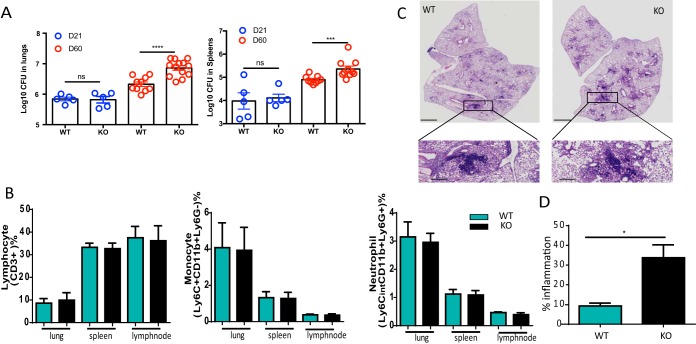
*Cd157* KO mice are more susceptible to chronic M. tuberculosis infection. Mice infected with strain H37Rv were sacrificed at 21 days and 60 days postinfection, and the lung and spleen were collected for assessing CD157 expression, M. tuberculosis bacterial load, or histopathology. (A) Bacterial burden in the lungs and spleens of WT mice and *Cd157* KO mice was assessed in the tissue homogenates. (B) The lungs, spleens, and mediastinal lymph nodes were collected, and a single cell suspension was prepared for cell surface staining. The percentages of lymphcyte (CD3^+^), monocytes (Ly6C^+^ CD11b^+^ Ly6G^−^), and neutrophils (Ly6Cint CD11b^+^ Ly6G^+^) in lungs, spleens, and mediastinal lymph nodes from *Cd157* KO and WT mice were determined. (C) Histopathological analysis of lung sections from WT and *Cd157* KO mice infected with M. tuberculosis. Sections were stained with hematoxylin and eosin (H&E). Images of H&E staining were captured using a NanoZoomer digital pathology system (Hamamatsu Photonics). The bars represent 1,000 μm. Outlined areas in the main images define the enlarged regions (magnification, ×10). (D) The percentage of inflammation was determined as the ratio of inflammation area within the whole section of lung tissue area using Nanozoomer 2.0 software (Hamamatsu Photonics). The bar graphs show the percentage of inflammation foci in the lungs from WT and *Cd157* KO mice. One-way ANOVA Newman-Keuls multiple comparison test (B) or unpaired *t* test (A and D) were used. Experiments were repeated at least twice, and the data represent the means ± SEM. Statistical significance: *, *P* < 0.05; ***, *P* < 0.001; ****, *P* < 0.0001; ns, not significant.

We then took advantage of *Cd157* knockout (KO) mice to further dissect the role of CD157 in TB pathogenesis. We found that compared to wild-type (WT) mice, *Cd157* KO mice showed increased bacterial burden in the lung and spleen at 60 days postinfection (Fig. 2A), indicating that deficiency of *Cd157* increases host susceptibility to TB. Intriguingly, despite the well-appreciated role of CD157 in regulating human monocyte migration, we found no difference in monocyte and macrophage infiltration into the lung, spleen, and mediastinal lymph node (MLN) between infected *Cd157* KO and WT mice, suggesting that deficiency of *Cd157* does not impair lymphocyte and monocyte recruitment in response to M. tuberculosis infection (Fig. 2B). Similar results were observed in neutrophils (Fig. 2B).

Besides functioning in innate immunity, recruited monocytes also serve as antigen-presenting cells to prime adaptive immunity (5). We then measured M. tuberculosis antigen-specific T cells by flow cytometry to further investigate the effect of CD157 on modulating T-cell responses. We found that the frequency of both CD4 and CD8 T cells in the MLN and spleen are not altered in the absence of CD157 (see Fig. S1A and B in the supplemental material). We also found that the cytokine (gamma interferon [IFN-γ], tumor necrosis factor alpha [TNF-α], and interleukin 17 [IL-17])-producing cells (CD4 and CD8) stimulated with M. tuberculosis lysates from spleens and MLNs were not significantly different between *Cd157* KO and WT mice (Fig. S1A and B). Histological analysis indicates an increase of inflammation within the lungs of M. tuberculosis-infected *Cd157* KO mice compared to M. tuberculosis-infected WT mice, perhaps as a consequence of the higher bacterial load (Fig. 2C and D). Together, these results support the hypothesis that CD157 confers a protective role against M. tuberculosis infection but that this is unlikely to be by modulating migration of monocyte and altering T-cell immunity.

10.1128/mBio.01949-19.1 FIG S1 CD157 deficiency does not affect the adaptive immune response in mice infected with M. tuberculosis. *Cd157* KO and WT mice were sacrificed at 60 days postinfection with strain H37Rv. The spleens and MLNs were collected, and a single cell suspension was prepared for cell surface staining or cultured in the presence of M. tuberculosis (Mtb) lysates (10 μg/ml) for intracellular cytokine staining. The percentages of different cell subsets were determined by flow cytometry. (A) Percentages of CD4 cells producing IFN-γ, TNF-α, and IL-17 in the spleen and lymph node after short-term *in vitro* stimulation with Mtb lysate, determined by flow cytometry. (B) Percentages of CD8 cells producing IFN-γ, TNF-α, and IL-17 were determined by FACS. Unpaired, two-tail Student’s *t* test was used. The data are expressed as means ± SEM. Download FIG S1, PDF file, 0.2 MB. Copyright © 2019 Yang et al. 2019 Yang et al. This content is distributed under the terms of the Creative Commons Attribution 4.0 International license.

### *Cd157* deficiency impairs macrophage bactericidal capacity.

**FIG 3:**
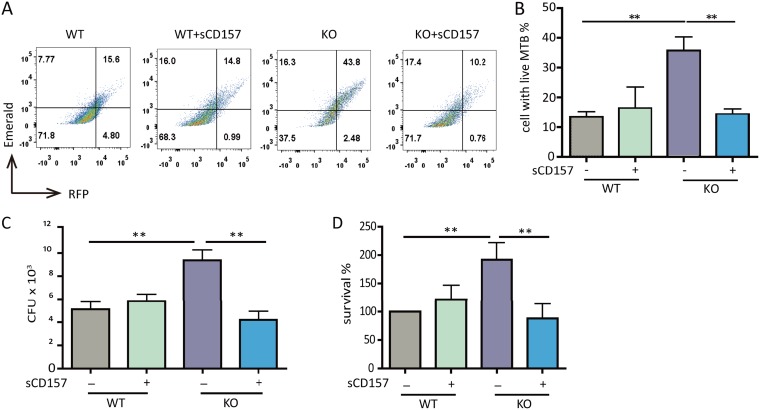
CD157 deficiency impairs macrophage bactericidal capacity. Peritoneal macrophages from WT mice and *Cd157* KO mice treated with sCD157 (+sCD157) (5 μg/ml) or not treated with sCD157 were infected with strain H37Ra harboring a dual-color reporter that comprises a constitutively green (Emerald) and a tetracycline-inducible red (TagRFP) fluorescent protein for 3 days. Tetracycline (500 ng/ml) was added 24 h before flow cytometry. Macrophages were then harvested and fixed with 4% PFA, and the percentage of cells with live M. tuberculosis (MTB) (red) were determined by FACS. The data are presented as representative dot plot data (A) and means plus SEM (B to D). Bacterial burden within peritoneal macrophages from WT mice and *Cd157* KO mice at 72 h postinfection with the H37Rv strain (MOI of 5) in the presence or absence of sCD157 (5 μg/ml). The data were presented as absolute number of CFU (C) or the percentage of survival relative to WT mock treatment (D). One-way ANOVA Newman-Keuls multiple comparison test (B, C, and D) was used. **, *P* ≤ 0.01.

Macrophages are critical effector cells of both innate and adaptive immunity in clearance of M. tuberculosis, besides their roles in regulating the adaptive immune response against M. tuberculosis (3, 19). Since T-cell immunity in *Cd157* KO mice was intact (Fig. S1A and B), we hypothesize that the increased TB susceptibility in *Cd157* KO mice might be due to impairment of macrophage bactericidal capacity against M. tuberculosis. To test this, we isolated peritoneal macrophages from WT and *Cd157* KO mice and tested their ability to kill M. tuberculosis by using an M. tuberculosis strain H37Ra harboring a dual-color reporter that comprises a constitutively expressed (Emerald, green) and a tetracycline-inducible (TagRFP, red) fluorescent protein, whose live or dead status within macrophage can be easily differentiated by fluorescence-activated cell sorting (FACS) (Fig. S2A). We found that the M. tuberculosis killing activity of *Cd157* KO macrophages was significantly impaired compared to WT macrophages (Fig. 3A and B), without affecting macrophage phagocytosis (Fig. S2B). Notably, such impairment was completely rescued upon exogenous application of sCD157 (Fig. 3A and B). These findings were further confirmed by CFU assays in M. tuberculosis H37Ra (Fig. 3C) or H37Rv (Fig. 3D)-infected *Cd157* KO and WT peritoneal macrophages (Fig. 3C and D). Collectively, these findings indicate that the ability of macrophages to kill M. tuberculosis is impaired by CD157 deficiency, which may contribute to increased TB susceptibility in *Cd157* KO mice.

10.1128/mBio.01949-19.2 FIG S2 (A) Macrophages were infected with strain H37Ra harboring a dual-color reporter. Peritoneal macrophages from WT mice were infected with H37Ra (WT-H37Ra) or H37Ra harboring a dual-color reporter that comprises a constitutively green (Emerald) and a tetracycline-inducible red (TagRFP) fluorescent protein for 3 days, with tetracycline (H37Ra+Atc) (500 ng/ml) or without tetracycline (H37Ra-Atc) for the last 24 h. Macrophages were then harvested and fixed with 4% PFA, and the percentages of cells with live M. tuberculosis (red) were determined by FACS. (B) M. tuberculosis (Mtb) uptake by macrophages from WT mice and CD157 KO mice at 4 h postinfection with the H37Rv (MOI = 5). Download FIG S2, PDF file, 0.1 MB. Copyright © 2019 Yang et al. 2019 Yang et al. This content is distributed under the terms of the Creative Commons Attribution 4.0 International license.

### Deficiency of *Cd157* impairs the bactericidal capacity of macrophages by inhibiting TLR2-dependent ROS production.

**FIG 4:**
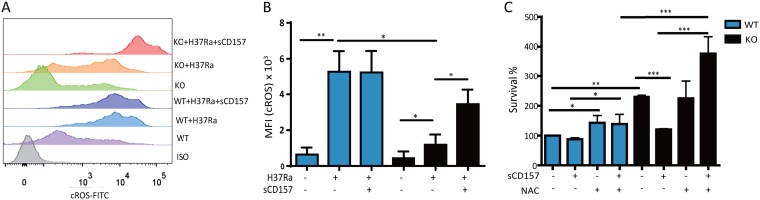
sCD157 inhibits M. tuberculosis intracellular growth in macrophages by modulating ROS production. Peritoneal macrophages from WT mice and *Cd157* KO mice treated with sCD157 (5 μg/ml) or not treated with sCD157 were infected with strain H37Ra (MOI of 5). The production of cROS after infection with strain H37Ra for 3 h was determined by FACS. FITC, fluorescein isothiocyanate. (A and B) The representative histogram (A) and averaged cROS mean fluorescence intensity (MFI) (B) were presented. (C) Peritoneal macrophages from WT mice and *Cd157* KO mice were infected with strain H37Ra (MOI of 5) in the presence or absence of NAC (200 μM). The bacterial burden was assessed 72 h after infection. One-way ANOVA Newman-Keuls multiple comparison test (B and C) was used. The data represent the means plus SEM. *, *P* ≤ 0.05, **, *P* ≤ 0.01, ***, *P* ≤ 0.001.

To further understand how CD157 deficiency impairs M. tuberculosis killing by macrophages, we tested the effect of CD157 on several canonical mechanisms of macrophage-mediated control of M. tuberculosis (20). These mechanisms included generation of reactive oxygen species (ROS) and nitric oxide (NO), as well as apoptosis and autophagy. There were no significant differences between *Cd157* KO and WT macrophages on NO production, apoptosis, and autophagy (Fig. S3A, B, and C). These data indicated that deficiency of *Cd157* does not affect these bactericidal functions in macrophages. We found that cytosolic ROS (cROS) production was significantly reduced in *Cd157* KO macrophages compared to WT macrophages after M. tuberculosis infection (Fig. S3D and Fig. 4A and B). Supplementing with exogenous sCD157 could rescue this impairment in *Cd157* KO macrophages but could not further enhance cROS production in WT infected macrophages (Fig. S3D and Fig. 4A and B).

10.1128/mBio.01949-19.3 FIG S3 CD157-induced M. tuberculosis infection resistance is not associated with NO production, cell autophagy, or cell apoptosis. Peritoneal macrophages from WT mice and *Cd157* KO mice were infected with M. tuberculosis (Mtb) H37Rv (MOI = 5) with or without sCD157 (5 μg/ml). Cells and supernatants were harvested at the indicated times and analyzed accordingly. (A) Cellular apoptosis was analyzed by PI/Annexin V flow cytometry after 72-h infection. (B) Autophagy was assessed by LC3β immunoblotting after 24-h infection. (C) NO production in supernatants was analyzed after 6-h and 24-h infection. (D) Peritoneal macrophages from WT mice and *Cd157* KO mice were infected with Mtb H37Rv (MOI = 5) with or without sCD157 (1, 2.5, or 5 μg/ml) for 3 h. Cells were then collected, and cROS was detected by flow cytometry. One-way ANOVA Newman-Keuls multiple comparison test (C and D) was used. The data represent means ± SEM. Download FIG S3, PDF file, 0.5 MB. Copyright © 2019 Yang et al. 2019 Yang et al. This content is distributed under the terms of the Creative Commons Attribution 4.0 International license.

10.1128/mBio.01949-19.4 FIG S4 CD157 interacts with TLR2 and PKCzeta. Peritoneal macrophages from WT mice infected with M. tuberculosis H37Ra (MOI = 5) for 12 h or not infected were lysed and immunoprecipitated with His-tagged CD157 protein. Immunoprecipitates were then immunoblotted for TLR2, PKCzeta, and His (His-CD157), respectively. Download FIG S4, PDF file, 0.5 MB. Copyright © 2019 Yang et al. 2019 Yang et al. This content is distributed under the terms of the Creative Commons Attribution 4.0 International license.

ROS is an efficient innate immune mechanism against M. tuberculosis (21). We further explored the role of ROS in CD157-mediated resistance to M. tuberculosis. To this end, we used a ROS scavenger, *N*-acetyl-l-cysteine (NAC), to deplete ROS during M. tuberculosis infection. As expected, we found that application of NAC abrogated the effect of sCD157 on rescuing the killing activity of *Cd157* KO macrophage (Fig. 4C). Taken together, we conclude that cROS is essential for the CD157-mediated M. tuberculosis killing ability of macrophages.

**FIG 5:**
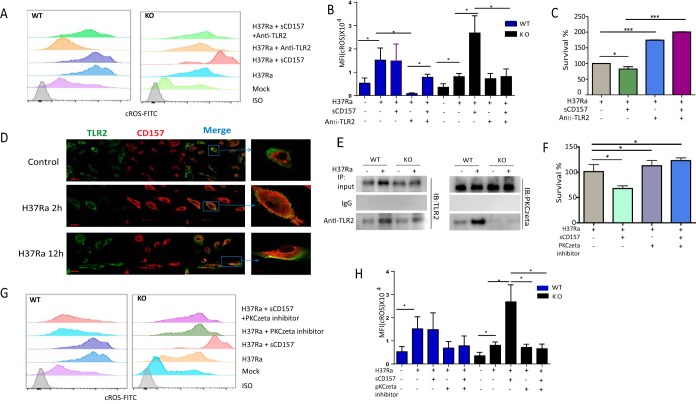
CD157 interacts with TLR2 and PKCzeta to enhance macrophage ROS production. Peritoneal macrophages from WT mice and *Cd157* KO mice infected with strain H37Ra (MOI of 5) for 3 h in the presence or absence of sCD157 (5 μg/ml) or anti-TLR2 (20 μg/ml). cROS production was determined by FACS. (A and B) The representative cROS production (A) and the averaged cROS MFI (B) were detected. (C) Peritoneal macrophages from *Cd157* KO mice were infected with strain H37Ra (MOI of 5) in the presence or absence of sCD157 (5 μg/ml) or anti-TLR2 (20 μg/ml). At 72 h postinfection, the number of CFU was determined. (D) Peritoneal macrophages from WT mice infected with strain H37Ra were analyzed for TLR2 (green) and CD157 (red) expression on macrophages by immunofluorescence staining. The bar represents 20 μm in small pictures at a magnification of ×80. The gray arrows indicate a region of colocalization. (E) Peritoneal macrophages from WT and KO mice infected with strain H37Ra (+) (MOI of 5) for 12 h or not infected with strain H37Ra (−) were lysed and immunoprecipitated (IP) with anti-TLR2 antibody. Immunoprecipitates were then immunoblotted (IB) for TLR2 and PKCzeta. (F) Peritoneal macrophages from *Cd157* KO mice were infected with M. tuberculosis H37Ra (MOI of 5) in the presence or absence of PKCzeta inhibitor (20 μM) or sCD157 (5 μg/ml). CFU was assessed 72 h postinfection. Peritoneal macrophages from WT mice and *Cd157* KO mice infected with strain H37Ra (MOI of 5) for 3 h in the presence or absence of sCD157 (5 μg/ml) or PKCzeta inhibitor (20 μM). cROS production was determined by FACS. (G and H) The representative cROS production (G) and the averaged cROS MFI (H) were detected. One-way ANOVA Newman-Keuls multiple comparison test (B, C, F, and H) was used. Values are means plus SEM of duplicate determinations of two independent experiments. *, *P* ≤ 0.05, ***, *P* ≤ 0.001.

Although CD157 is recognized as a receptor, the current knowledge on its natural ligands suggest that CD157 itself has no function to sense M. tuberculosis invasion. In contrast, numerous pattern recognition receptors (PRRs) can sense M. tuberculosis infection and initiate host responses against M. tuberculosis (22). Among these PRRs, Toll-like receptor 2 (TLR2) has an essential role in M. tuberculosis-induced ROS production by macrophages (21). We therefore investigated the potential role of TLR2 in CD157-mediated cROS production and host resistance to M. tuberculosis infection. In line with previous reports (21, 23), antibody-mediated TLR2 blockade significantly inhibited M. tuberculosis-induced cROS production in WT macrophages (Fig. 5A and B). In contrast, blocking TLR2 abrogated the effects of sCD157 on rescuing cROS production and M. tuberculosis killing activity of *Cd157* KO macrophages (Fig. 5A to C). Our data showed that sCD157 has a stronger effect on Cd157 KO macrophages for ROS production than on WT macrophages, and sCD157 could rescue the defect of M. tuberculosis killing only in *Cd157* KO macrophages and could not enhance the bactericidal activity of WT macrophages. Interestingly, we observed that sCD157 could enhance ROS production in TLR2 blockage WT macrophages (Fig. 5B), indicating that the cross talk of sCD157 and TLR2 is very complex in WT macrophages. The roles of sCD157 on ROS production in infected WT macrophages with anti-TLR2 need to be further defined. Together, these data indicate that CD157 is involved in TLR2-dependent ROS production in *Cd157* KO macrophages.

### CD157 participates in M. tuberculosis-induced TLR2-dependent ROS production via a TLR2 and PKCzeta interaction.

TLR2 activation upon sensing mycobacteria can initiate a cascade of responses, including inflammation, autophagy, metabolism, and even cell death (24–26). For examples, CD36 and TLR2 cooperate in signaling compartmentalization within membrane rafts and divert the host response signaling through peroxisome proliferator-activated receptor γ (PPARγ)-dependent and NF-κB-independent pathways to increase macrophage lipid accumulation (27). On the other hand, protein kinase Czeta (PKCzeta) is specifically recruited upon activation by a 19-kDa lipoprotein from M. tuberculosis to interact with TLR2 and regulate ROS and inflammatory cytokine production, such as TNF-α (28). Therefore, we hypothesized that CD157 might enhance the compartmentalization of TLR2 and PKCzeta, and therefore selectively drive cROS production. To test this hypothesis, we first investigated the protein interactions between TLR2 and CD157 in mouse peritoneal macrophages following M. tuberculosis infection. Our immunofluorescence staining showed that CD157 is distributed both on the membrane and in the cytoplasm (Fig. 5D) and colocalized with TLR2 upon M. tuberculosis infection (Fig. 5D). We next determined whether CD157 interacts with TLR2 and PKCzeta by coimmunoprecipitation (Co-IP). We detected TLR2 and PKCzeta in anti-His (His-CD157) immunoprecipitates by using the exogenous histidine-tagged CD157 (His-CD157) in WT macrophages, with increased binding capacity to CD157 in M. tuberculosis-infected macrophages (Fig. S4). In addition, we further confirmed that CD157 enhanced TLR2 and PKCzeta interaction, as we observed the increased binding capacity of TLR2 and PKCzeta in WT macrophages, compared to *Cd157* KO macrophages (Fig. 5E). Such interaction was functionally validated using a PKCzeta inhibitor, application of which significantly abrogated the effect of CD157 on M. tuberculosis killing activity and ROS production (Fig. 5F to H).

We then aimed to define the downstream signaling pathway involved in CD157-mediated ROS production. Here, we investigated the involvement of extracellular signal-regulated kinase (ERK) and the NF-κB signaling pathway, which are important for TLR2-dependent ROS production. M. tuberculosis infection efficiently activated NF-κB signaling, as indicated by significant enhancement of canonical NF-κB-dependent cytokines, such as TNF-α and IL-6 expression (Fig. S5A). Similarly, we found that ERK phosphorylation was quickly increased upon M. tuberculosis infection (Fig. S5B), indicating activation of ERK signaling by M. tuberculosis. However, both ERK and NF-κB activation remained intact in M. tuberculosis-infected *Cd157* KO macrophages (Fig. S5B and C). Together, these data suggest that NF-κB and ERK signaling are unaffected by CD157 deficiency.

10.1128/mBio.01949-19.5 FIG S5 CD157 does not enhance ROS production via an ERK/NFκB-pathway. (A) Peritoneal macrophages from WT mice and *Cd157* KO mice infected with H37Ra (MOI = 5) in the presence of sCD157 (5 μg/ml) for 24 h or in the absence of sCD157 for 24 h. The relative expression of TNF-α and IL-6 was determined by quantitative PCR. (B) Peritoneal macrophages from WT mice and *Cd157* KO mice infected with H37Ra (MOI = 5). ERK and phosphorylated ERK (pERK) expression in peritoneal macrophages were detected by Western blotting (WB) at 0, 5, 15, and 30 min after H37Ra infection. (C) Peritoneal macrophages from WT mice and *Cd157* KO mice infected with strain H37Ra (MOI = 5). P65 and phosphorylated P65 (p-p65) expression in peritoneal macrophages were detected by WB at 20 min after H37Ra infection. One-way ANOVA Newman-Keuls multiple comparison test (A) was used. Download FIG S5, PDF file, 1.3 MB. Copyright © 2019 Yang et al. 2019 Yang et al. This content is distributed under the terms of the Creative Commons Attribution 4.0 International license.

### The sCD157 level correlates with host immunity against TB.

**FIG 6:**
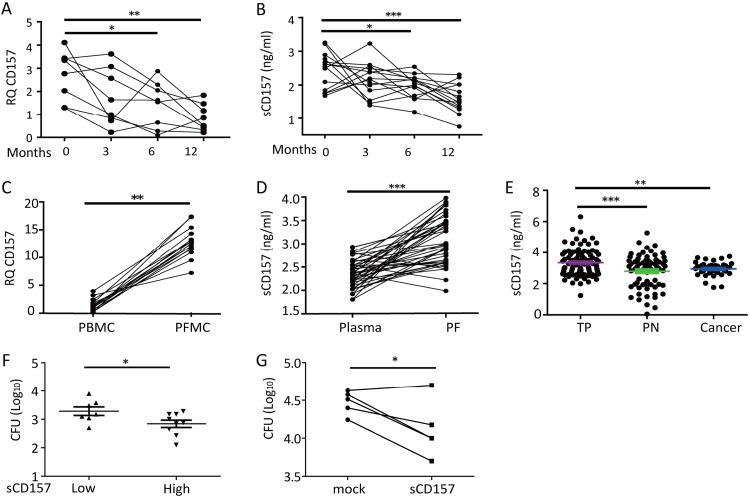
CD157 expression decreases after anti-TB treatment and is associated with bactericidal activity of MDMs. Whole blood or plasma was collected before (0 month) and different times (3, 6, and 12 months) after initiation of anti-TB treatment. (A) *Cd157* expression in whole blood from patients with TB (*n* = 8) during anti-TB treatment was determined by qPCR. (B) The plasma sCD157 levels in patients with TB (*n* = 14) during anti-TB treatment were determined by ELISA. (C) *Cd157* expression in paired PBMCs and PFMCs from patients with TP (*n* = 15) was determined by qPCR. (D) The concentrations of sCD157 in paired plasma and pleural fluid (PF) samples from patients with TP (*n* = 48) were determined by ELISA. (E) The concentrations of sCD157 in pleural fluid from patients with TP (*n* = 94), pneumonia (PN) (*n* = 73), and lung cancer (*n* = 37) were determined. (F) Monocyte-derived macrophages (MDMs) from HCs (*n* = 16) were divided into two groups according to the expression of plasma sCD157 (low, sCD15 < 1,736 pg/ml; high, sCD15 ≥ 1,736 pg/ml). MDMs were infected with M. tuberculosis H37Ra (MOI of 5) for 72 h, and the numbers of CFU were determined and compared. (G) MDMs from HCs (*n* = 5) were infected with strain H37Ra (MOI of 5) in the presence or absence (mock) of exogenous sCD157 (5 μg/ml) for 72 h, and the bacterial burdens within MDMs were determined by CFU counting. One-way ANOVA Newman-Keuls multiple comparison test (A, B, and E), Wilcoxon matched paired test (C, D, and G), or unpaired, two-tail Student’s *t* test (F) were used. *, *P* ≤ 0.05, **, *P* ≤ 0.01, ***, *P* ≤ 0.001.

Finally, we investigated whether the effects of sCD157 in restoring the host response to M. tuberculosis in *Cd157* KO mice can be translated to human TB. We found that *Cd157* expression in whole blood from TB patients gradually decreases after successful anti-TB chemotherapy (Fig. 6A). A similar downward trend was found for sCD157 in the plasma from the same patients (Fig. 6B). In addition, both mRNA and protein levels of CD157 were significantly higher in pleural fluid mononuclear cells (PFMCs) than in parallel PBMCs from patients with tuberculous pleurisy (Fig. 6C and D), suggesting that sCD157 may help fight against TB at the site of infection. In support of this proposal, sCD157 levels in pleural fluid from TB patients were significantly higher than those from patients with non-TB diseases, which included pneumonia and lung cancer (Fig. 6E). Moreover, we found that sCD157 plasma levels are inversely associated with M. tuberculosis killing activity of monocyte-derived macrophages (MDMs) *in vitro*. We first divided healthy individuals into two groups (sCD157 low and sCD157 high) based on the average level of sCD157 in plasma (Fig. 1E) and then compared the M. tuberculosis killing activity of MDMs in these two groups. We found that MDMs from individuals with low sCD157 levels (<1,736 pg/ml) were less efficient in controlling M. tuberculosis replication than MDMs from individuals with high sCD157 levels (≥1,736 pg/ml) (Fig. 6F), implying that baseline sCD157 is a potential biomarker to predict susceptibility to TB. Finally, exogenous application of sCD157 to MDMs from individuals with low sCD157 levels significantly enhanced M. tuberculosis killing activity (Fig. 6G). These data suggest that sCD157 levels may be assessed in patients as a potential precision medicine approach for TB. Those with low baseline sCD157 may benefit from exogenous sCD157 treatment to control M. tuberculosis infection.

## DISCUSSION

CD157 is expressed on leukocytes and physiologically mediates monocyte and neutrophil migration (8, 11, 12). Here, we found that CD157 expression was higher in the patients with TB compared to all other patient groups. In agreement with our human data, CD157 was significantly increased in the mouse lung during M. tuberculosis infection, suggesting that CD157 might have an important role in TB pathogenesis. Using *Cd157* KO mice, we confirmed that CD157 does indeed have a protective role against M. tuberculosis infection. Unexpectedly, this protective role of CD157 is not dependent on its regulatory role on monocyte and neutrophil migration but instead on its contribution to TLR2-dependent ROS production in *Cd157* KO macrophages after M. tuberculosis infection. This novel function of CD157 occurs via enhancing the interaction between TLR2 and PKCzeta. Impaired immune protection as a result of CD157 deficiency in macrophages can be rescued by exogenous application of sCD157, implicating a potential utility of sCD157 for host-directed therapy against TB in patients with CD157 deficiency.

In contrast to the important role of CD157 in transendothelial migration and adhesion of monocyte and neutrophils in a homeostasis state (11, 12), we found no difference in the numbers of monocytes, macrophages, and neutrophils in the lungs of M. tuberculosis-infected WT and *Cd157* KO mice. This finding suggests that the contribution of CD157 to cell migration during M. tuberculosis infection is limited. While we are uncertain of the exact reasons underlying such a difference, one explanation may be that the impairment of CD157 function in cell migration might be compensated for by increased chemokine-receptor interactions induced by M. tuberculosis. For example, CCR2 and its ligands MCP-1, MCP-2, and MCP-3, which recruit monocytes to the lung, are significantly increased upon M. tuberculosis infection (19, 29, 30). Similarly, mice lacking CD38, a sister molecule of CD157 with 33% homology in amino acid sequence, are highly susceptible to Mycobacterium avium without impaired immune cell recruitment to the lung (8). These *Cd38* KO mice have high numbers of granulomas compared to their WT counterparts (31). Once they arrive at the site of M. tuberculosis infection, i.e., the lymph nodes and the lung, the monocytes can still differentiate into dendritic cells and macrophages, through which both adaptive and innate immunity are augmented to contain M. tuberculosis infection (5). Unlike *Cd38* KO, which increases susceptibility by modulating T-cell differentiation and polarization, *Cd157* KO does not markedly affect adaptive T-cell immunity, probably due to differential distribution of these two molecules among immune cells (8, 9).

ROS production by macrophages is important for building host resistance against mycobacterial infection. Patients with chronic granulomatous diseases typically have a genetic deficiency in the ROS-producing phagocyte NADPH oxidase NOX2 and are highly susceptible to mycobacterial infection (32). Germ-line mutations in CYBB, the human gene encoding the gp91^Phox^ subunit of the phagocyte NOX2, selectively impairs macrophagic but not neutrophilic ROS production; affected patients also show high susceptibility to mycobacterial infection (33). Several studies have demonstrated that M. tuberculosis induces NADPH activation and ROS production in a TLR2-dependent manner (21, 28, 34, 35). Consequently, impairments to this biological process due to a deficiency in TLR2 and its downstream molecule, MyD88, or in enzymes required for ROS production, significantly increase susceptibility to chronic M. tuberculosis infection (21, 33, 36, 37). Conversely, M. tuberculosis-secreted virulent factors, such as ESAT-6, inhibit TLR2 signaling to avoid macrophage-mediated cell death (38).

We found that CD157 deficiency significantly reduces TLR2-dependent ROS, but not NO production, and therefore impairs the M. tuberculosis killing ability of macrophages. This novel function of CD157 is achieved through its interaction with TLR2 and PKCzeta, a critical molecule for M. tuberculosis-derived 19-kDa lipoprotein-induced TLR2-dependent ROS production (28). However, CD157 deficiency had no effect on the roles of ROS in regulating cytokine secretion, cell apoptosis, and cell autophagy during M. tuberculosis infection. In support of this, we found that ERK and NF-κB signaling were unaffected in *Cd157* KO macrophages upon M. tuberculosis infection. One explanation is that the level of ROS produced in *Cd157* KO macrophages is still efficiently induced downstream of the ERK and NF-κB signal, as both *CD157* KO and WT macrophages efficiently activate these signals. Although we cannot exclude the possible involvement of ERK and NF-κB signaling in TLR2-CD157-PKCzeta-ROS production, the defect in ROS production caused by *Cd157* KO may be an isolated process that leads to increased susceptibility to TB without affecting classical innate immunity. A recent study reported that PKCzeta may directly activate P47^phox^ to generate ROS (39). Therefore, it is possible that CD157 diverts TLR2 functions to this novel ROS production pathway through TLR2-CD157-PKCzeta cooperation.

An interesting finding of our study is that sCD157 efficiently rescues the impaired ROS production caused by *Cd157* KO, in which the mice are deficient for both the membrane-bound and secreted forms of CD157. Although we are uncertain whether the function of CD157 in regulating ROS production is mediated by membrane-bound CD157, sCD157, or both *in vivo*, our macrophage experiments suggest that both cell-associated and soluble CD157 can modulate macrophage functions during M. tuberculosis infection *in vitro*. However, sCD157 has a stronger effect on Cd157 KO macrophages for ROS production than on WT macrophages, and sCD157 could rescue the defect of M. tuberculosis killing only in KO macrophages, not in WT macrophages. Interestingly, we observed that sCD157 could enhance ROS production in M. tuberculosis-infected WT macrophages with anti-TLR2. One explanation may be that the two forms of CD157 act through different mechanisms or pathways, even cross-regulate each other in WT macrophages. The mechanism of sCD157 and TLR2 on ROS production in M. tuberculosis-infected WT macrophages need to be further investigated.

Importantly, we have identified that sCD157 levels correlate with host immunity against TB in humans. sCD157 is enriched in the pleural fluid of TB patients, and its concentration is significantly higher in TB patients than in non-TB controls. Therefore, our data suggest that sCD157 levels may be a useful indicator of the ability of macrophages to kill M. tuberculosis. Application of sCD157 might be an effective strategy for host-directed therapy against TB in those with insufficient CD157 production.

## MATERIALS AND METHODS

### Ethics statement.

This study was conducted according to the principles expressed in the Declaration of Helsinki. Ethical approval was obtained from the Research Ethics Committee of Shenzhen Third People’s Hospital. All participants provided written informed consent for sample collection and subsequent analyses. All experimental procedures on mice were performed in accordance with the Regulations for the Administration of Affairs Concerning Experimental Animals approved by the State Council of People’s Republic of China. The animal experimental protocols were approved by the Animal Research Ethics Committee of Shenzhen Third People’s Hospital.

### Subjects and clinical sample collection.

The three patient cohorts used in this study were recruited from the Shenzhen Third People’s Hospital and the First Affiliated Hospital, Shenzhen University. The demographic characteristics of these study populations are provided in Table S1 in the supplemental material. Cohort I included 55 cases of healthy control (HC), 46 cases of latent tuberculosis infections (LTBI), 54 cases of pulmonary tuberculosis (TB), and 38 cases of pneumonia. Cohort II included 94 cases of TB pleurisy (TP). The non-TB pleurisy group included 37 cases of malignant effusions (lung cancer) and 73 cases of pneumonia. At the time of enrollment, all TB patients had no record of prior TB disease or anti-TB chemotherapy, except those (*n* = 14) being monitored for the kinetic change of CD157 during anti-TB chemotherapy. A total of 16 cases of HC were included in cohort III.

10.1128/mBio.01949-19.6 TABLE S1 Demographic characteristics of study populations. Download Table S1, DOCX file, 0.01 MB. Copyright © 2019 Yang et al. 2019 Yang et al. This content is distributed under the terms of the Creative Commons Attribution 4.0 International license.

Diagnosis of active TB was based on clinical symptoms, chest radiography, and microscopy for acid-fast bacilli (AFB), sputum and/or bronchoalveolar lavage fluid (BALF) M. tuberculosis culture, and response to anti-TB chemotherapy (40). Healthy controls with normal chest radiographic findings and without a clinical history of TB were recruited. M. tuberculosis-specific interferon gamma release assays (IGRAs) were used to differentiate individuals with LTBI from HC without infection as described previously (40). Individuals with LTBI were recruited from household contacts of active TB patients, and they all had no evidence of disease or history of TB. TP was diagnosed based on pleural fluid and/or biopsy specimen cultures or by observation of granulomatous inflammation in pleural biopsy tissue as described previously (41). The diagnoses of pneumonia and lung cancer were based on the following. (i) Lavage fluid or sputum cultures were M. tuberculosis negative during clinical follow-up. (ii) New infiltration and clinical signs on chest radiography were evident and completely resolved after treatment with the appropriate antibiotics. (iii) Viral pathogens were not detected. (iv) Pathological findings supported the diagnosis (40, 41).

### Isolation of PBMCs and PFMCs.

Heparinized whole-blood samples were collected by venipuncture from all participants, and pleural fluid samples were collected from patients by pleural effusion. Peripheral blood mononuclear cells (PBMCs) were obtained by gradient separation of whole blood as previously described (40). Pleural fluid mononuclear cells (PFMCs) and supernatants were separated by centrifugation of up to 50 ml pleural fluid at 300 × *g* for 5 min. Isolated PBMCs and PFMCs were immediately used for RNA extraction or stored at –150°C. The plasma and supernatant from the pleural fluid were stored at –80°C for CD157 assays.

### RNA extraction and quantitative reverse transcription-PCR (qRT-PCR).

Total RNA was extracted from human whole-blood samples, PBMCs, or mouse lung homogenate using a RNeasy minikit (Qiagen) following the manufacturer’s instructions. Purified RNA was reverse transcribed to cDNA using PrimeScript RT reagent kit (TaKaRa). Quantitative PCR (qPCR) was performed using SYBR Green PCR Master Mix (TaKaRa) following a standard protocol. The relative mRNA expression of the target genes was calculated by comparison with the glyceraldehyde-3-phosphate dehydrogenase (GAPDH) housekeeping gene using the 2^−△△Ct^ method (42). The primers were as follows: human CD157 sense, 5′-ACAGCACCCATCCTGACTGT-3′; human CD157 antisense, 5′-GAAGCCAGCACCAGAAAGAG-3′; mouse CD157 sense, 5′-CGCCAACTTTGCCATACAGC-3′; mouse CD157 antisense, 5′-CTCTTCATTAACCTCTCCAGGC-3′.

### Murine TB model.

C57BL/6 mice and *Cd157* knockout (KO) mice, which have been backcrossed eight times to C57BL/6 mice (43) aged 6 to 8 weeks were used for M. tuberculosis infection. M. tuberculosis infection was performed using the virulent H37Rv strain. C57BL/6 and *Cd157* KO mice were infected using a Glas-Col inhalation exposure system (Glas-Col, USA) to deliver ∼450 CFU of M. tuberculosis per mouse. At the indicated time points after infection, mice were sacrificed, and the organs were aseptically excised, individually homogenized, and subsequently plated on 7H11-OADC (oleic acid-albumin-dextrose-catalase) agar (BD Biosciences) for 3 weeks to count the CFU.

### Immunohistochemistry.

Segments of lung tissue isolated from mice infected with M. tuberculosis were fixed in 10% buffered formalin (Sigma-Aldrich) and embedded in paraffin. Histologic sections were stained with hematoxylin and eosin (H&E) for pathological evaluation. Images of the whole microscope slide were captured using a NanoZoomer digital pathology system (Hamamatsu Photonics). For immunohistochemistry, lung tissue from human TB patients who received surgery were collected and frozen in liquid nitrogen. Serial 5-mm sections of paraffin-embedded tissue were used for immunohistochemical staining. Briefly, tissues were fixed with acetone-chloroform for 3 min and then incubated for 2 h with anti-CD157 (ab137718; Abcam). Primary antibodies were detected using a biotinylated secondary antibody system (PolinkDS-MR kit; Golden Bridge International Co.) following the manufacturer’s instructions. Images were captured using a NanoZoomer digital pathology system (Hamamatsu Photonics).

### Macrophage isolation.

Peritoneal macrophages were isolated as previously described (44). Wild-type (WT) C57BL/6 and *Cd157* KO mice were injected intraperitoneally with 2 ml of 3% thioglycolate medium (catalog no. T9032; Sigma-Aldrich). After 3 days, cells were harvested by peritoneal lavage with cold phosphate-buffered saline (PBS) and allowed to adhere for 2 h. Nonadherent cells were removed by washing twice with PBS. The remaining adherent cells were cultured in Dulbecco modified Eagle medium (DMEM) (10% fetal bovine serum [FBS]) and used for M. tuberculosis infection. PBMCs were isolated from whole-blood samples from healthy control (HC) subjects and cultured in RPMI 1640 medium supplemented with 10% FBS (10% FBS-RPMI 1640 medium) for 2 h. The supernatant was removed. and adhered cells were cultured in 10% FBS-RPMI 1640 medium in the presence of human 10 ng/ml granulocyte-macrophage colony-stimulating factor (GM-CSF) (catalog no. BMS324; eBioscience) for 7 or 8 days to differentiate monocyte-derived macrophages (MDMs).

### Macrophage infections.

Peritoneal macrophages from WT and *Cd157* KO mice were infected with M. tuberculosis H37Rv and H37Ra strains, respectively. After 4 h of incubation, noninternalized bacteria were washed twice with PBS and incubated in fresh complete medium with or without 5 μg/ml soluble CD157 (sCD157) (catalog no. 50319-M08H; Sino Biological Inc.). Cells were lysed with 0.1% SDS at 0 and 72 h after infection and then plated on 7H11-OADC plates in serial dilutions. The numbers of CFU were counted after 3 weeks. In some experiments, macrophages were pretreated with sCD157 (5 μg/ml) (catalog no. 50319-M08H; Sino Biological), *N*-acetyl-l-cysteine (NAC) (200 μM) (catalog no. S0077; Beyotime), PKCzeta inhibitor (PKCzeta pseudo-substrate inhibitor, myristoylated, 20 μM) (sc-397537; Santa Cruz) or anti-TLR2 (20 μg/ml) (catalog no. 121802; Biolegend) (45). For flow cytometry, peritoneal macrophages pretreated with or without sCD157 were infected with an M. tuberculosis strain harboring a dual-color reporter that comprises a constitutively (Emerald, greed) and a tetracycline-inducible (TagRFP, red) fluorescent protein (kindly provided by Christopher M. Sassetti, University of Massachusetts Medical School). At day 3 postinfection, tetracycline (500 ng/ml) (catalog no. 64-75-5; MedChemExpress) was added to the medium, and macrophages were harvested after 24 h of incubation, fixed with 4% paraformaldehyde (PFA), and analyzed using a BD FACSCanto II (BD Biosciences). MDMs (4 × 10^5^/well) were cultured in six-well plates and infected with strain H37Ra at a multiplicity of infection (MOI) of 5. After incubation at 37°C for 4 h, cells were washed twice with PBS and incubated with fresh RPMI 1640 medium supplemented with 10% FBS. Cells were lysed with 0.1% SDS at 72 h postinfection. Serial dilutions were plated, and the number of CFU was counted after 3 weeks.

### Confocal microscopy.

Peritoneal macrophages were infected with strain H37Ra as described above. At different time points, cells were collected, fixed with acetone-chloroform for 3 min, and then incubated for 2 h with anti-TLR2 (ab11864: Abcam) and anti-CD157 (ab208442; Abcam). The secondary antibodies used were Alexa Fluor 555-labeled goat anti-rabbit (ab150078; Abcam) and Alexa Fluor 647-labeled goat anti-rat antibody (A-21248T; Thermo Scientific). Negative controls were obtained by using isotype-matched primary antibodies. Images of the cells were captured under a Zeiss LSM700 laser scanning confocal microscope.

### Measurement of cytosolic reactive oxygen species (cROS).

Peritoneal macrophages from WT and *Cd157* KO mice pretreated with or without sCD157 were infected with strain H37Ra at an MOI of 5. Cells were harvested at 3 h postinfection, washed with Hanks’ balanced salt solution (HBSS), and resuspended in HBSS with 5 mM CMH2DCFDA (catalog no. C6827; Invitrogen). Cells were incubated at 37°C with 5% CO_2_ for 15 min, washed with HBSS, and then analyzed using a BD FACSCanto II.

### Coimmunoprecipitation.

Peritoneal macrophages were infected with strain H37Ra for 12 h. Cells were harvested after treatment in NETN buffer containing 20 mM Tris-HCl (pH 7.5), 150 mM NaCl, 1 mM EDTA, 0.5% Nonidet P-40, and protease inhibitor mixture. Anti-TLR2 antibody (ab209217; Abcam) and IgG were added to the cell lysates and incubated at 4°C overnight. After SDS-PAGE, immunoblots on polyvinylidene difluoride (PVDF) membranes were blocked with 5% nonfat milk in PBST (PBS with 0.05% Tween 20). Primary antibodies against target proteins in 5% milk in PBST were incubated with the membranes at 4°C overnight, and after extensive washing with PBST, secondary antibodies in 5% milk in PBST were incubated with the membranes for 1 h at room temperature. Anti-TLR2 and anti-PKCzeta (sc-393218; Santa Cruz) antibodies were purchased from Abcam and Santa Cruz Biotechnology, respectively.

### Flow cytometry.

Fresh whole-blood samples (200 μl each) from HC and TB subjects or PFMCs from TP subjects were used for flow cytometric analysis of CD157 expression. Briefly, erythrocytes were lysed in lysing solution (catalog no. 347691; BD Biosciences), and then the samples were stained with anti-CD14 (catalog no. 347493; BD Biosciences), anti-CD3 (catalog no. 557749; BD Biosciences), and anti-CD157 (catalog no. 12-1579-42; eBioscience). At least 0.2 million cells were acquired for analysis using FACSDiva software (BD Biosciences).

### Cd157 ELISA.

The levels of CD157 in plasma and pleural fluid were determined by enzyme-linked immunosorbent assays (ELISAs) following the manufacturer’s instructions (E1551Hu; USCN Life Science). CD157 levels in mouse serum were detected by ELISA following the manufacturer’s instructions (catalog no. NS-E10161; NovaTeinBio).

### Statistical analyses.

All statistical tests were performed in GraphPad Prism version 7.0 (GraphPad Software Inc.). The one-way analysis of variance (ANOVA) Newman-Keuls multiple comparison test was used to compare differences among multiple groups. An unpaired *t* test was used to analyze the difference between two groups. The Wilcoxon matched paired test was used to analyze the difference between paired samples. Differences were considered significant when *P *was *<*0.05.
