# Supplementary figures and images for: CD157 Confers Host Resistance to Mycobacterium tuberculosis via TLR2-CD157-PKCzeta-Induced Reactive Oxygen Species Production
Source: mBio. 2019 Aug 27;10(4):e01949-19. doi: 10.1128/mBio.01949-19 (PMC6712401; doi:10.1128/mBio.01949-19)

Mtb lysates stimulated

A

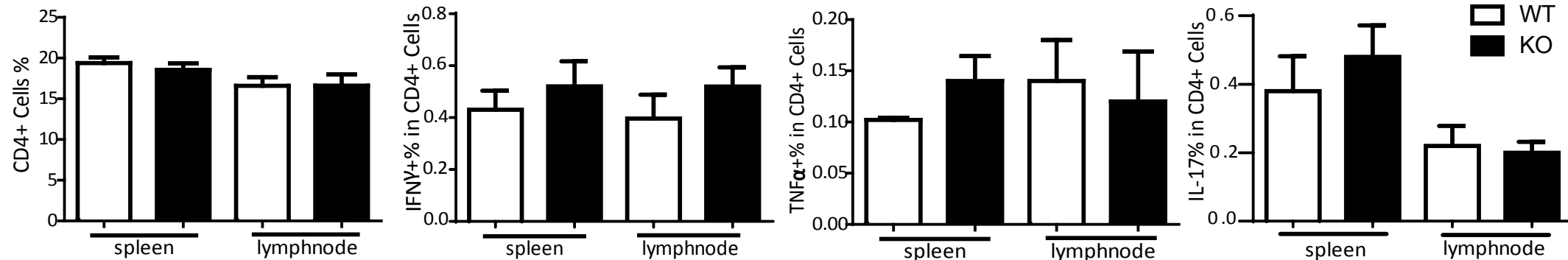

B

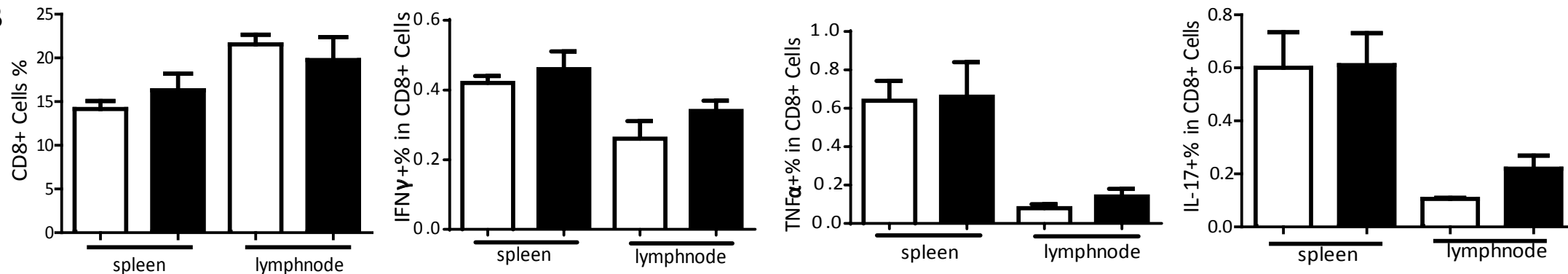

Supplement: FIG S1 [file mBio.01949-19-sf001.pdf]

A

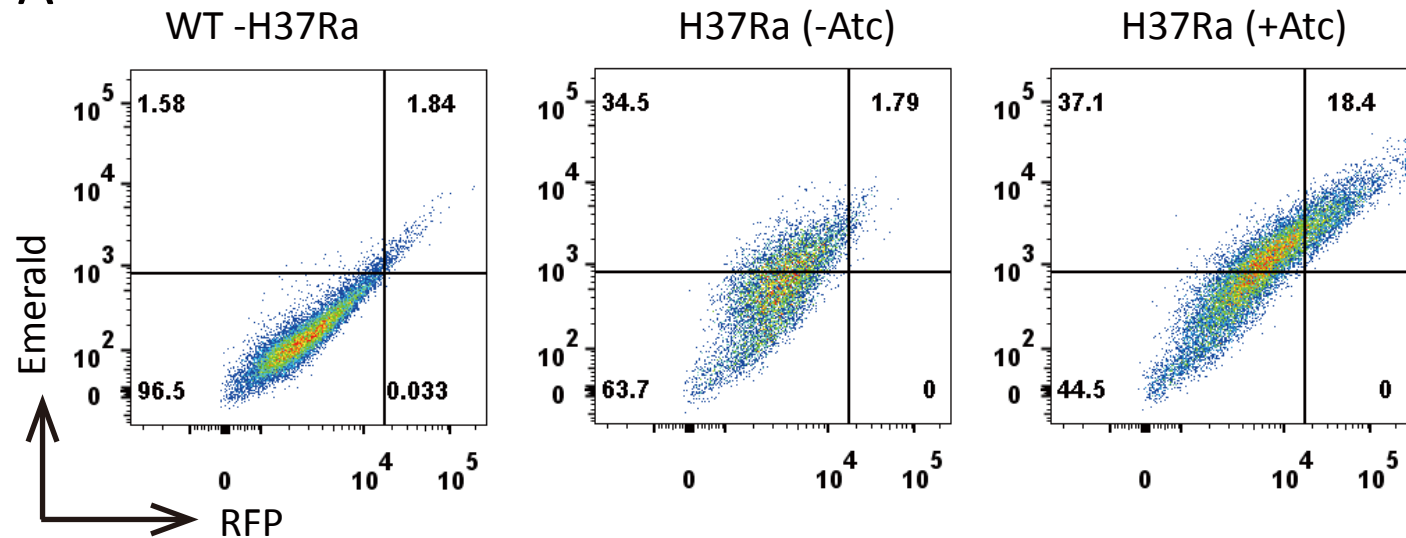

B

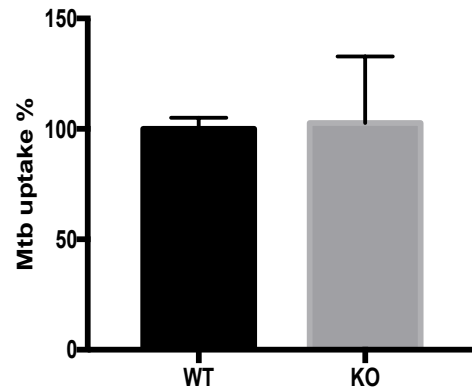

Supplement: FIG S2 [file mBio.01949-19-sf002.pdf]

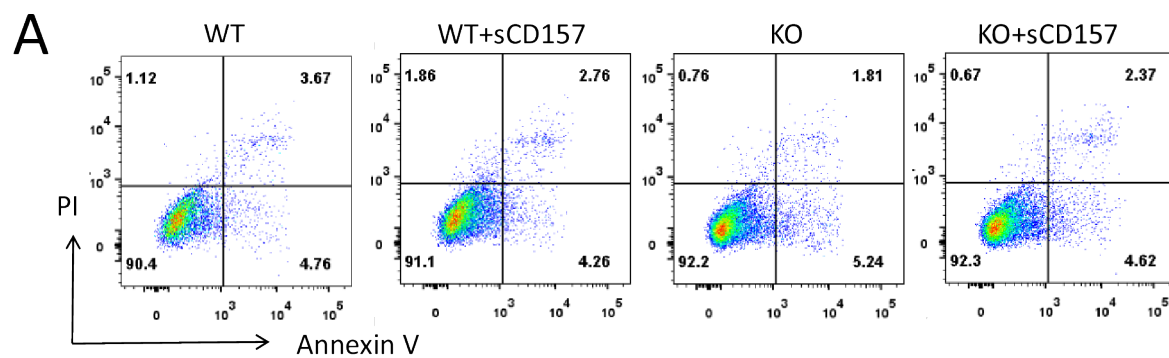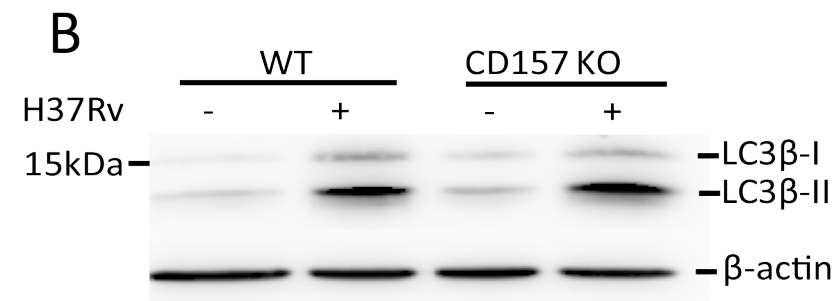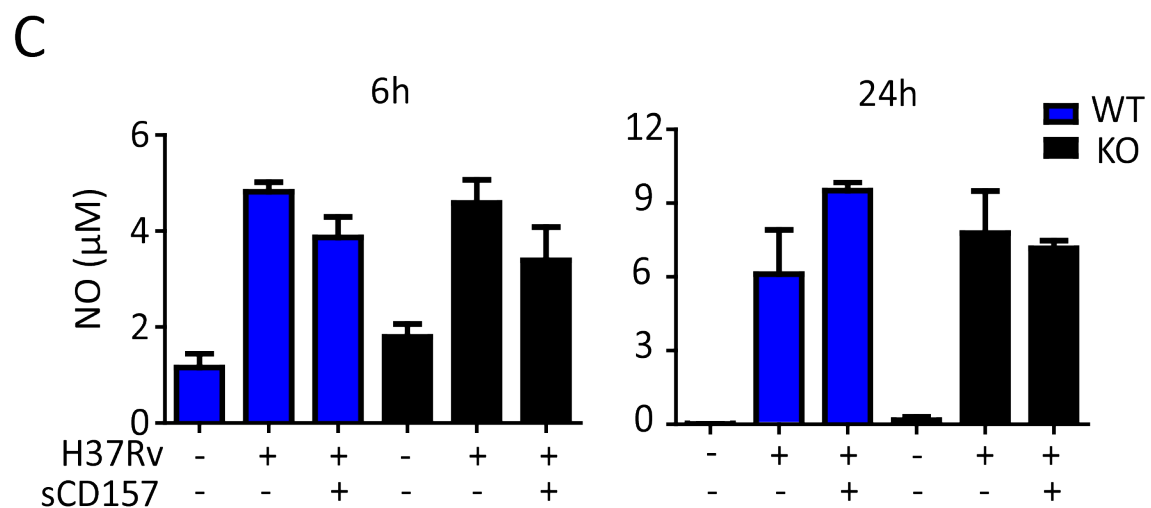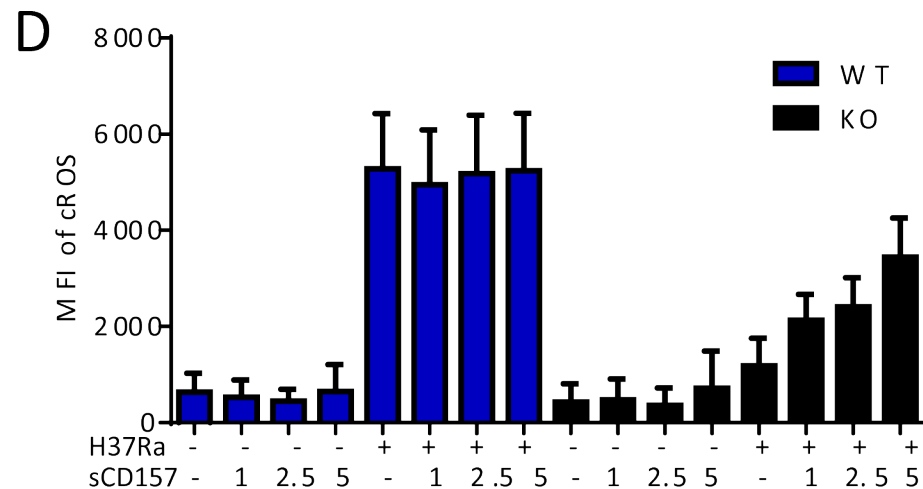

Supplement: FIG S3 [file mBio.01949-19-sf003.pdf]

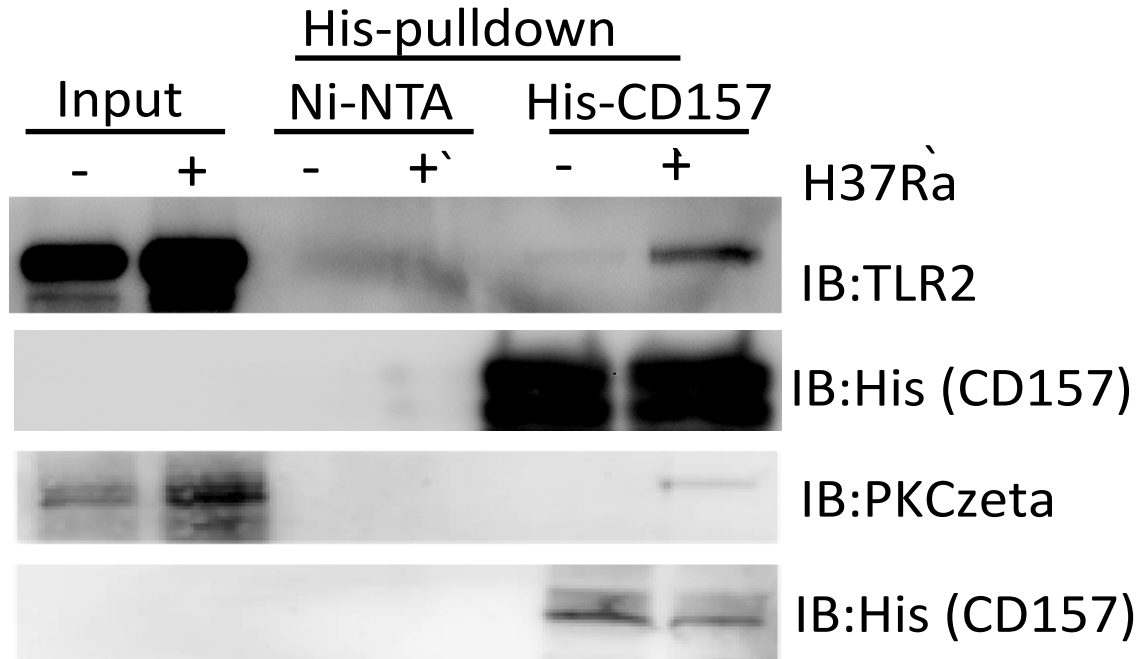

Supplement: FIG S4 [file mBio.01949-19-sf004.pdf]

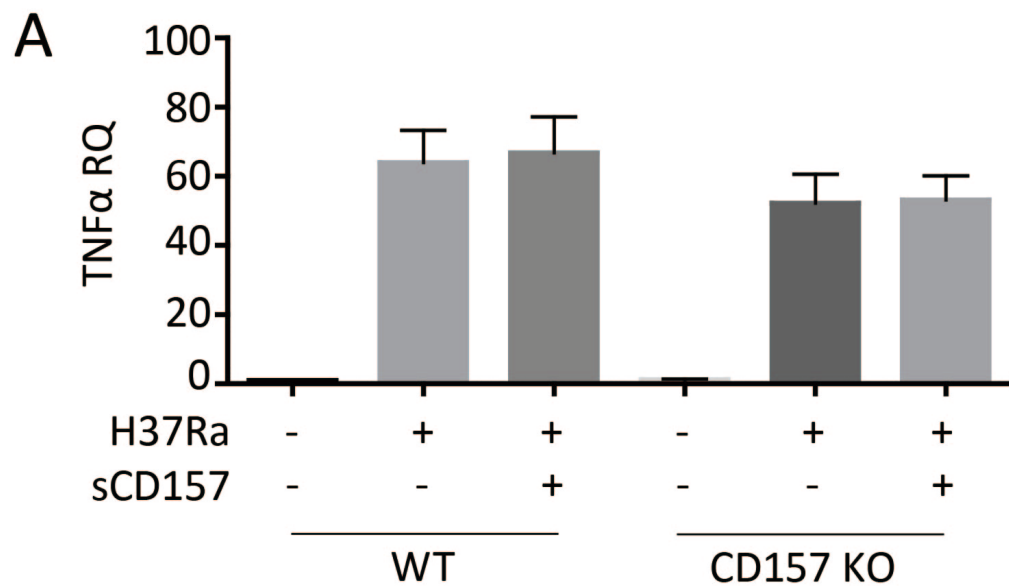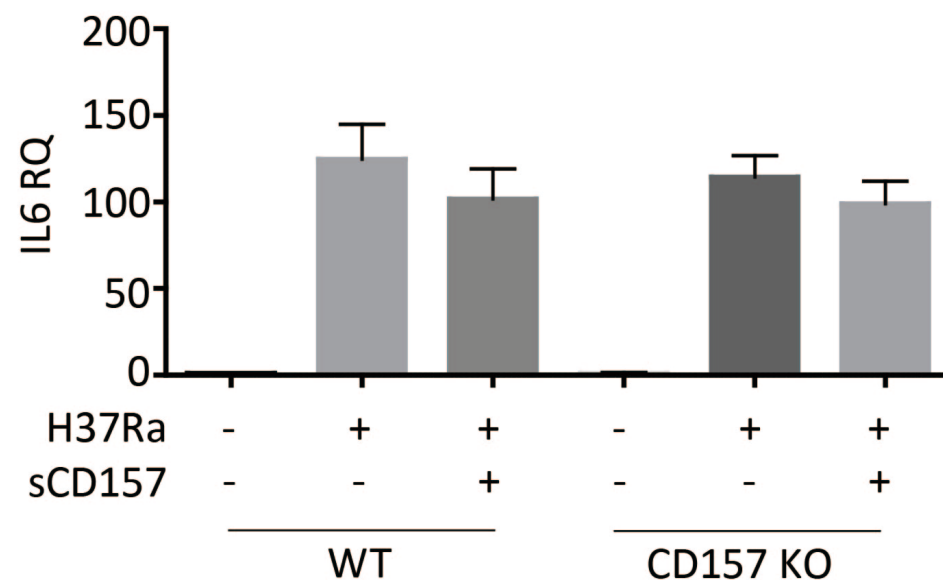

**B**

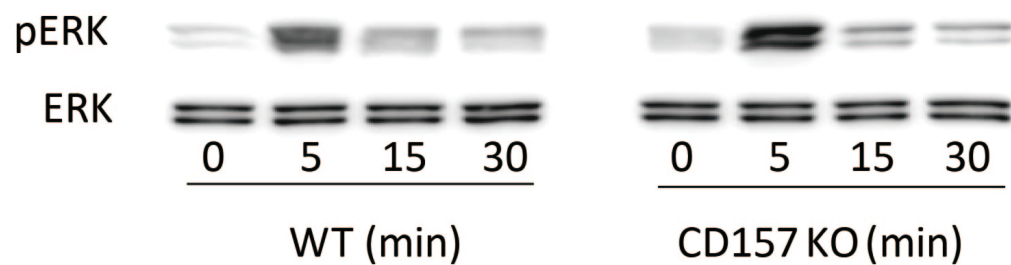

**C**

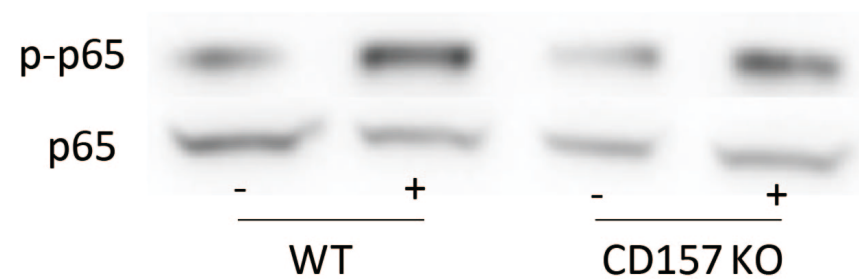

Supplement: FIG S5 [file mBio.01949-19-sf005.pdf]
