# Supplementary material for: CD157 Confers Host Resistance to Mycobacterium tuberculosis via TLR2-CD157-PKCzeta-Induced Reactive Oxygen Species Production
Source: mBio. 2019 Aug 27;10(4):e01949-19. doi: 10.1128/mBio.01949-19 (PMC6712401; doi:10.1128/mBio.01949-19)
Supplement: TABLE S1 [file mBio.01949-19-st001.docx]

**Supplemental table 1 Demographic characteristics of study populations**

| **Study Group** | **Subject** | **Case No.** | **Sex (M/F)** | **Age (Years)** | **type of sample** |
| --- | --- | --- | --- | --- | --- |
| I | HC^a^ | 55 | 15/30 | 31.16 ±0.935 | whole blood or plasma |
|  | LTBI | 46 | 14/32 | 32.50±1.105 | whole blood or plasma |
|  | TB^b^ | 54 | 36/18 | 36.63±1.360 | whole blood or plasma or lung tissue |
|  | Pneumonia | 38 | 22/16 | 40.62±2.107 | whole blood or plasma |
| II | TP^c^ | 94 | 20/74 | 36.13±1.698 | pleural fluid or PBMC or plasma or PFMC |
|  | Pneumonia | 73 | 21/52 | 43.42±2.398 | pleural fluid |
|  | lung cancer | 37 | 12/25 | 57.11±2.212 | pleural fluid |
| III | HC^d^ | 16 | 11//5 | 28.5±1.118 | plasma and PBMC |

a CD157 expression in whole blood was detected by flow cytometry in 21/55 HC cases and 20/54 TB cases. The levels of sCD157 in the plasma samples were detected by ELISA in 46/55 HC cases,46/46 LTBI cases, 40/54 TB cases, and 17/38 pneumonia cases.
b Dynamic changes in CD157 expression was assessed in 8/54 cases in whole blood and 14/54 cases before and after anti-TB treatment. CD157 expression in lung were detected by IHC in 3/54 case in lung tissue.
c CD157 levels in peripheral blood mononuclear cells (PBMC) and parallel pleural fluid mononuclear cells (PFMC) were compared in 15/94 TP cases; the levels of sCD157 in the pleural fluid and parallel plasma samples were compared in 48/94 TP cases.
d Monocyte derived macrophages were treated with or without exogenous sCD157 and were infected with Mtb H37Ra for colony forming unit assays in 5/16 HC, whose sCD157 expression in plasma was <1736 pg/ml
